# Experimental study on tissue-engineered urethral graft for repairing urethral defects in a rabbit model

**DOI:** 10.3389/fbioe.2025.1711552

**Published:** 2025-12-19

**Authors:** Aodi Jiang, Yu Tian, Chengyang Sun, Ying Lian, Hongfeng Zhai

**Affiliations:** 1 Department of Plastic and Aesthetic Surgery, Henan Provincial People’s Hospital, Zhengzhou, China; 2 Department of Breast Surgery, Henan Provincial People’s Hospital, Zhengzhou, China; 3 Department of Plastic and Aesthetic Surgery, People’s Hospital of Henan University, People’s Hospital of Zhengzhou University, Henan Provincial People’s Hospital, Zhengzhou, China

**Keywords:** acellular dermal matrix, tissue engineering, urethral mucosal epithelial cells, urethral reconstruction, urethral stricture

## Abstract

**Objective:**

Tissue engineering represents a promising alternative for managing severe urethral defects. This study aimed to construct a tissue-engineered urethral graft by combining autologous urethral mucosal epithelial cells with an acellular dermal matrix (ADM) and evaluate its efficacy in tubularized repair of long-segment urethral defects in rabbits.

**Methods:**

Autologous urethral mucosal epithelial cells were harvested and seeded onto ADM scaffolds to fabricate tissue-engineered grafts. Cell adhesion, proliferation, and viability on the scaffolds were assessed using scanning electron microscopy (SEM), CCK-8 assay, and histological analysis. Thirty-six rabbits were randomly divided into three groups (n = 12 per group): Group A (control), where defects were repaired by end-to-end anastomosis; Group B (ADM-only), where defects were repaired using a tubularized ADM graft; and Group C (cell–ADM composite), where defects were repaired using a tubularized cell-seeded ADM graft. Radiographic, gross, and histological evaluations were performed 12 weeks post-surgery.

**Results:**

The ADM scaffold demonstrated good biocompatibility. The incidence of postoperative complications was 100% in Group A, 66.67% in Group B, and 8.33% in Group C. Statistically significant differences were observed between Group A and Group B, as well as between Group B and Group C (P < 0.05). Urethroscopy, imaging, and histological examinations revealed superior regenerative outcomes in Group C compared to Group B.

**Conclusion:**

The tubularized urethral mucosal epithelial cell–ADM composite graft is a feasible and effective strategy for repairing long-segment urethral defects in rabbits, exhibiting significantly better outcomes than ADM alone. Thus, this tissue-engineered construct represents an ideal alternative for long-segment urethral reconstruction.

## 1 Introduction

Urethral injuries result from diverse etiologies, including congenital anomalies, inflammation, trauma, and tumors. Such injuries frequently lead to urethral strictures, which can subsequently trigger severe complications such as bladder stones, urethral fistulas, sepsis, and even renal failure (Gelman and Furr, 2020). The incidence of these conditions increases with age (Campbell and Broghammer, 2025). The formation of a urethral stricture is the central pathological event in this cascade.

The core pathological feature of urethral stricture is fibrosis of the extracellular matrix within the corpus spongiosum (Cavalcanti et al., 2007). This process initiates with microvascular injury, which induces tissue hypoxia and inflammation, activating the transformation of fibroblasts into myofibroblasts. This transformation promotes the excessive deposition and aberrant cross-linking of stiff, thick type I collagen and mediates extracellular matrix contraction, ultimately resulting in loss of urethral compliance and pathological luminal narrowing.

Given the irreversibility of these pathological changes, surgical repair remains the sole therapeutic option for long-segment urethral strictures. The reconstruction and repair of the urethra present significant challenges in the fields of plastic surgery, urology, and pediatric surgery. Autologous tissue substitutes, such as skin grafts or buccal mucosa grafts, are currently the clinical mainstay (Cheng et al., 2018). However, this gold-standard approach has notable limitations, including donor site morbidity, technical complexity, and the risk of postoperative complications such as recurrent stricture (Xu et al., 2015).

To overcome the constraints of autologous grafts, tissue engineering offers a novel strategy for constructing biomimetic grafts (Guo and Ma, 2018). A cornerstone of this approach is the use of biological scaffolds to facilitate cell migration and tissue regeneration. A variety of materials are available for urethral repair, primarily including acellular matrices (e.g., small intestinal submucosa (SIS), bladder acellular matrix (BAM), acellular dermal matrix (ADM)), natural polymers (e.g., collagen, silk fibroin, bacterial cellulose), and synthetic polymers (e.g., polylactic acid (PLA), polyglycolic acid (PGA), PLGA) (Jin et al., 2025). Synthetic materials often provoke chronic inflammation due to their degradation products. In contrast, natural acellular materials demonstrate greater clinical potential owing to their superior biocompatibility and richness in growth factors.

Despite the diversity of available materials, the repair strategy itself is evolving from a “cell-free” to a “cell-based” paradigm. Research on acellular grafts began in the early 2000s, but they are primarily suitable for short-segment defects. For more challenging long-segment defects, acellular matrices often lead to repair failure due to delayed vascularization and uncontrolled fibrosis. Consequently, developing a cell-seeded graft that effectively repairs long-segment defects while balancing procedural simplicity and cost-effectiveness has become a critical bottleneck in the field awaiting a breakthrough.

**FIGURE 1:**
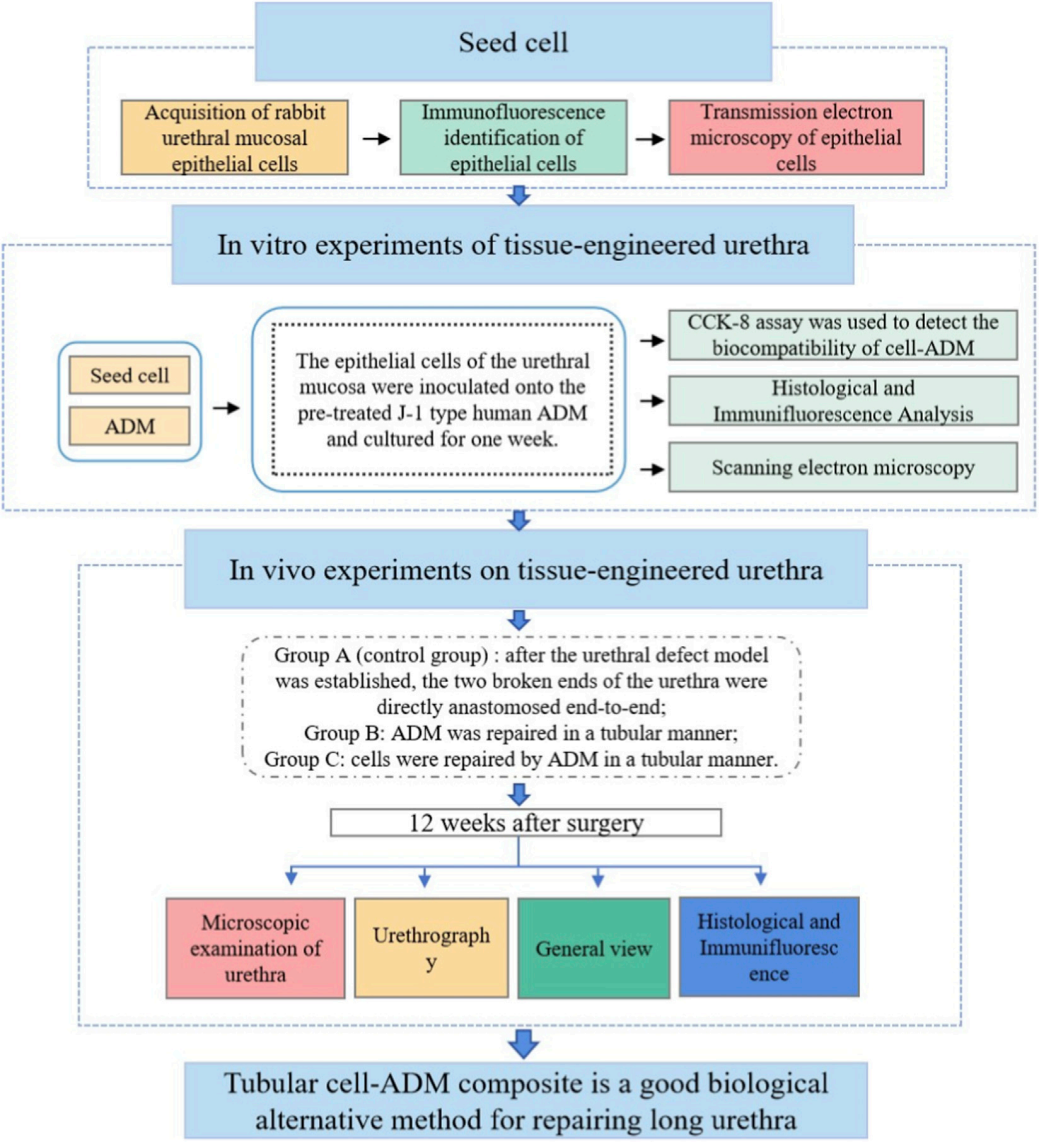
Schematic illustration of the study design.

To address this challenge directly, this study developed a novel tubularized graft. We pre-seeded autologous rabbit urethral epithelial cells onto an acellular dermal matrix (ADM). This cell-matrix composite strategy aims to establish a proactive biological microenvironment, offering the advantage of overcoming the inherent limitations of traditional onlay grafts (only suitable for ventral repair) or simple tubularized material grafts (lacking bioactivity) (Wang et al., 2025). We hypothesize that this strategy will provide a more stable and clinically translatable solution for the functional regeneration of circumferential, long-segment urethral defects. A schematic overview of the study design is presented in Figure 1.

## 2 Methods

### 2.1 Isolation, immunofluorescence characterization, and transmission electron microscopy of urethral mucosal epithelial cells

Healthy adult male New Zealand white rabbits were anesthetized, and the perineal region was aseptically prepared with povidone-iodine (Beijing Lianchang Hygiene Disinfection Products Co., Ltd., China) and draped with sterile surgical towels. An incision was made in the anterior urethral wall to obtain a 4 × 5 mm mucosal tissue segment. The tissue was rinsed 2–3 times with PBS (Solarbio, China) containing penicillin and streptomycin. The mucosal layer was separated by submucosal injection of normal saline followed by blunt dissection, minced into small fragments, and digested with 2.4 U/mL Dispase II (Gibco, United States) at 37 °C for 1.5 h. After PBS washing, the epithelial layer was finely minced and further digested in 3 mL of 0.25% trypsin (Gibco, United States) at 37 °C for 5 min. Digestion was terminated by adding fetal bovine serum (Gibco, United States). The cell suspension was filtered through a 200-μm nylon mesh and centrifuged at 1,000 rpm for 5 min. The pellet was washed twice with complete culture medium and resuspended for counting.

To remove contaminating fibroblasts, a differential attachment procedure was performed: cells were allowed to settle in culture flasks for 30 min at 37 °C, after which the medium was transferred to a new flask. This process was repeated three times to obtain a purified population of urethral mucosal epithelial cells.

Immunofluorescence staining was performed on cells cultured to 50%–60% confluence on poly-L-lysine-coated coverslips. After PBS rinsing, cells were fixed with 4% paraformaldehyde (4 °C, 20 min), permeabilized with 0.5% Triton X-100 (Beyotime, Shanghai), and blocked with normal goat serum. Incubation with anti-PCK antibody (Bioss, Beijing) was carried out overnight at 4 °C, followed by a Cy3-conjugated secondary antibody (Proteintech, United States) at 37 °C for 30 min. Nuclei were stained with DAPI (Beyotime), and images were acquired using a BX53 fluorescence microscope (Olympus, Japan).

Cells were fixed with 2.5% glutaraldehyde (Solarbio, China) and pelleted by centrifugation. Post-fixation was performed with 1% osmium tetroxide (Solarbio, China) at 4 °C for 1–2 h. Samples were then dehydrated through a graded ethanol series and embedded in resin. Ultrathin sections (60 nm) were prepared using an ultramicrotome (RMC, United States), followed by staining with uranyl acetate and lead citrate. Cellular ultrastructure was examined using a JEM-1200EX transmission electron microscope (TEM) (JEOL, Japan) at 80.0 kV.

### 2.2 Fabrication of cell–ADM constructs

The commercially available J-I type human acellular allogeneic dermal matrix (Beijing Jieya Lifeng Biological Technology Co., Ltd., China) was sterilized by ^60^Co irradiation and placed in sterile culture dishes. The matrix was immersed in serum-free medium, with the medium changed every 24 h for a total of three changes. Twenty-four hours prior to cell seeding, the medium was removed, and 50 μL of 1% qualified fetal bovine serum was applied evenly to the surface of the ADM. The coated matrix was pre-conditioned in a cell culture incubator for 24 h prior to use.

Third-passage urethral mucosal epithelial cells were harvested at 70%–80% confluence and resuspended at a density of 4 × 10^7^ cells/mL. A volume of 50 μL of cell suspension was added per 1 cm^2^ of the pre-conditioned J-I ADM. After 5 days of culture, the ADM was carefully transferred onto a sterile stainless-steel grid using sterile ophthalmic forceps and maintained at the air-liquid interface for 1 week to stimulate epithelial growth and stratification. Biocompatibility between the cells and ADM was assessed using the CCK-8 assay. Samples were harvested for hematoxylin and eosin (H&E) staining, immunohistochemistry (IHC), and scanning electron microscopy.

### 2.3 CCK-8 assay for cell–ADM biocompatibility

Sterile ADM scaffolds were trimmed into 20 pieces (4.0 × 4.0 × 0.5 mm) and placed in a 96-well plate, with 20 additional wells serving as blank controls without ADM. Third-passage rabbit urethral mucosal epithelial cells in good growth condition were seeded at a density of 1 × 10^3^ cells/well (100 μL) into both the ADM and control wells, with four replicates per group. The plate was incubated at 37 °C in a humidified atmosphere with 5% CO_2_. On days 1, 3, 5, 7, and 9 of culture, 10 μL of CCK-8 solution (Beyotime Biotechnology Co., Ltd., Shanghai, China) was added to each well according to the manufacturer’s instructions. After 3 h of incubation, the absorbance at 450 nm was measured using a microplate reader (Thermo Fisher Scientific Inc., Waltham, MA, United States).

### 2.4 Scanning electron microscope (SEM)

For scanning electron microscopy evaluation, ADM and cell-ADM constructs were fixed overnight in 2.5% glutaraldehyde at 4 °C and post-fixed with 1% osmium tetroxide for 2 h. After PBS (Solarbio, Beijing) washing, specimens were dehydrated through a graded ethanol series, critical-point dried, and sputter-coated with a thin gold layer. Samples were examined using a scanning electron microscope (Olympus, Japan) operating at 3.00 kV (scale bars: 50 μm, 10 μm).

### 2.5 Establishment of urethral defect model and surgical repair

A total of 36 healthy adult male New Zealand white rabbits were randomly divided into three groups (n = 12 per group, Table 1). All animal procedures were reviewed and approved by the Institutional Animal Care and Use Committee (IACUC) of Henan Xinuogu Biotechnology Co., Ltd. (Ethical Approval No.: ZXNG-2023100901), and were conducted in accordance with the NIH Guide for the Care and Use of Laboratory Animals. The grouping was as follows: Group A (Control): After defect creation, the urethral ends were directly re-anastomosed in an end-to-end fashion.Group B (ADM-only): The defect was repaired using a tubularized ADM graft.Group C (Cell–ADM composite): The defect was repaired using a tubularized graft composed of urethral mucosal epithelial cells seeded on ADM.

**TABLE 1:** Animal experiment grouping.

Group	Description	Animals (*n*)
A	Following urethral defect model establishment, direct end-to-end anastomosis was performed to restore urethral continuity	12
B	Tissue repair utilizing ADM alone	12
C	Surgical reconstruction via composite grafting of urethral mucosal epithelial cells and ADM	12

**FIGURE 2:**
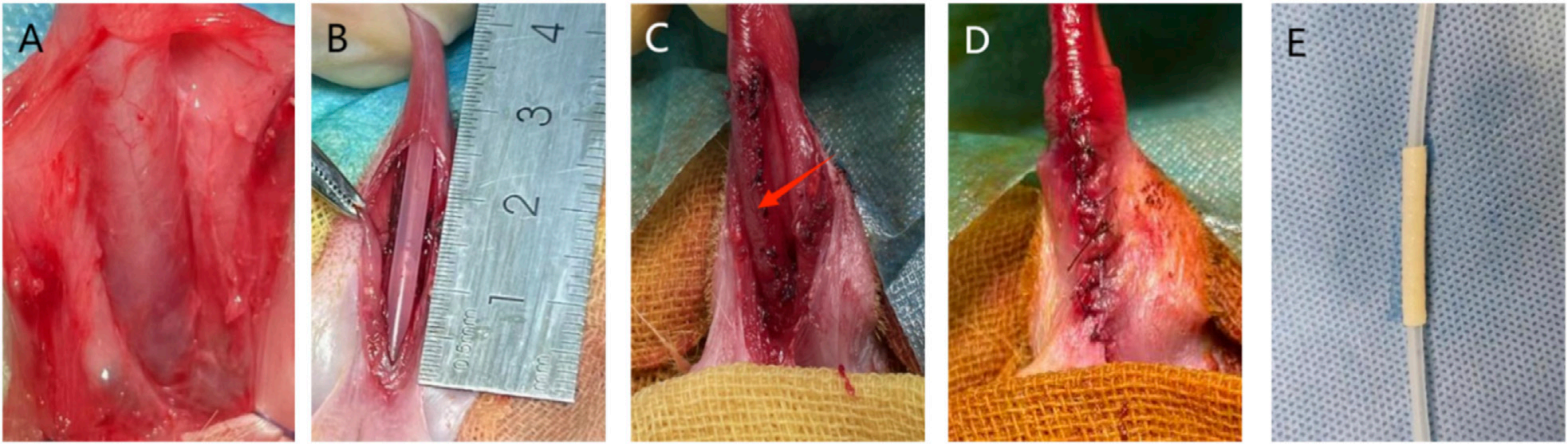
Surgical procedure for repair of the urethral defect in a rabbit model. **(A)** Exposure of the urethra after ventral midline incision and dissection. **(B)** Creation of a 2.5 cm-long urethral defect segment. **(C)** Repair of the defect using a tubularized graft (The red arrow points to the repair material). **(D)** Closure of tissue layers by suturing, with an indwelling 8Fr silicone catheter in place for urinary drainage and support. **(E)** Fabrication of a Tubular Urethral Mode.

Under general anesthesia and sterile conditions, the perineum and preputial orifice were shaved. Rabbits were positioned supine, and the perineal region was shaved and aseptically prepared by applying a 10% povidone-iodine solution (Beijing Lianchang, China) in a circular motion from the center to the periphery, repeated three times. The solution was allowed to air dry for complete antisepsis. The surgical site was draped with sterile surgical towels to establish an aseptic field. An 8Fr silicone catheter with side holes was inserted into the bladder to drain residual urine, and the bladder was irrigated with gentamicin (North China Pharmaceutical Co., Ltd., China)-containing saline until the effluent was clear. A traction suture (4–0 silk) was placed at the glans penis to straighten the penis and stabilize the catheter. A 3.0 cm longitudinal ventral incision was made starting 1 cm distal to the urethral meatus. The skin and subcutaneous tissues were incised sequentially. The ventral urethral corpus spongiosum was carefully dissected to expose the mucosal layer using ophthalmic scissors (Storz, Germany). The mid-urethra was completely mobilized via blunt dissection with micro-forceps and mosquito hemostats (Storz, Germany). A 2.5 cm segment of the mid-urethra was excised. Hemostasis was achieved by electrocautery, and the surgical site was irrigated with gentamicin-saline, successfully establishing the urethral defect model (Figure 2).

In Group A, the two urethral ends were directly anastomosed. The subcutaneous tissue and skin were then sutured sequentially to reconstruct the penile shaft. In Group B, a pre-hydrated ADM graft was trimmed such that its internal diameter slightly exceeded that of the catheter. It was tubularized around an 8Fr silicone catheter using a continuous 6–0 absorbable suture to form a neourethra approximately 2.7 cm in length (Figure 2E). The graft was anastomosed to both ends of the native urethra and sutured to the corpora cavernosa for stabilization. Wound closure was performed as in Group A.In Group C, the surgical procedure was identical to Group B, except that the graft consisted of the pre-cultured autologous urethral mucosal epithelial cell–ADM composite.In all groups, the catheter was securely fixed using the 4–0 traction suture, trimmed 1.0 cm distal to the urethral meatus, and retained for 10 days.

Postoperative Care: Elizabethan collars were fitted on all rabbits to prevent self-trauma to the surgical site or catheter. The wound was disinfected with povidone-iodine and redressed twice daily for 7 days. The catheter was flushed daily for 7 days with gentamicin-saline to maintain patency and reduce infection risk. Penicillin G sodium (North China Pharmaceutical Co., Ltd., China; 800,000 IU) was administered intramuscularly twice daily for 3 days for systemic antibiotic prophylaxis.

### 2.6 Urethroscopy and urethrography

Prior to specimen harvest, all animals underwent urethroscopic and radiological evaluations under general anesthesia. Following satisfactory anesthesia, rabbits were placed in a supine position with limbs and head secured. The urethra was examined using a rigid urethroscope after proper lubrication to assess mucosal regeneration, surface smoothness, and potential stricture formation. Subsequently, retrograde urethrography was performed by injecting meglumine diatrizoate contrast medium (Shanghai Xudong Haipu Pharmaceutical Co., Ltd., China) through a catheter secured at the urethral meatus. Urethral continuity and the presence of complications, such as fistula or stricture, were assessed under fluoroscopy.

### 2.7 Gross and histological examination

At 12 weeks post-operation, after completing the examinations, animals were euthanized. The perineal region was shaved, disinfected, and draped. The surgical site was exposed by dissection for gross examination of the urethral mucosa regarding smoothness, color, texture, scar contracture, and defects. Tissue samples were harvested and processed for H&E staining to evaluate epithelial regeneration. Immunohistochemical staining was performed using primary antibodies against pan-cytokeratin (PCK) and CD31 (both from Bioss, China) to identify urothelial cells and assess microvessel density, respectively, in the regenerated tissue.

### 2.8 Histological and immunifluorescence

For histological evaluation, both cell-ADM constructs and 12-week postoperative urethral tissues were processed through standard dehydration, clearing, and paraffin embedding. Sections (5 μm) were stained with H&E following established protocols.For immunofluorescence staining, after deparaffinization and rehydration, antigen retrieval was performed using Tris-EDTA buffer (pH 9.0). Sections were incubated with primary antibodies against (PCK) and CD31 at 4 °C overnight, followed by incubation with appropriate fluorescent-conjugated secondary antibodies. Nuclei were counterstained with DAPI, and all sections were examined under a fluorescence microscope (Olympus, Japan).

### 2.9 Statistical analysis

Categorical data were compared using Fisher’s exact test, with a significance level of α = 0.05. The positively stained areas for PCK and CD31 were quantified using ImageJ software and expressed as mean ± standard deviation (SD). Intergroup differences were analyzed by one-way analysis of variance (ANOVA). All statistical analyses were performed using SPSS version 25.0, and a *P* < 0.05 was considered statistically significant.

## 3 Results

### 3.1 Immunofluorescence and TEM characterization of urethral mucosal epithelial cells

**FIGURE 3:**
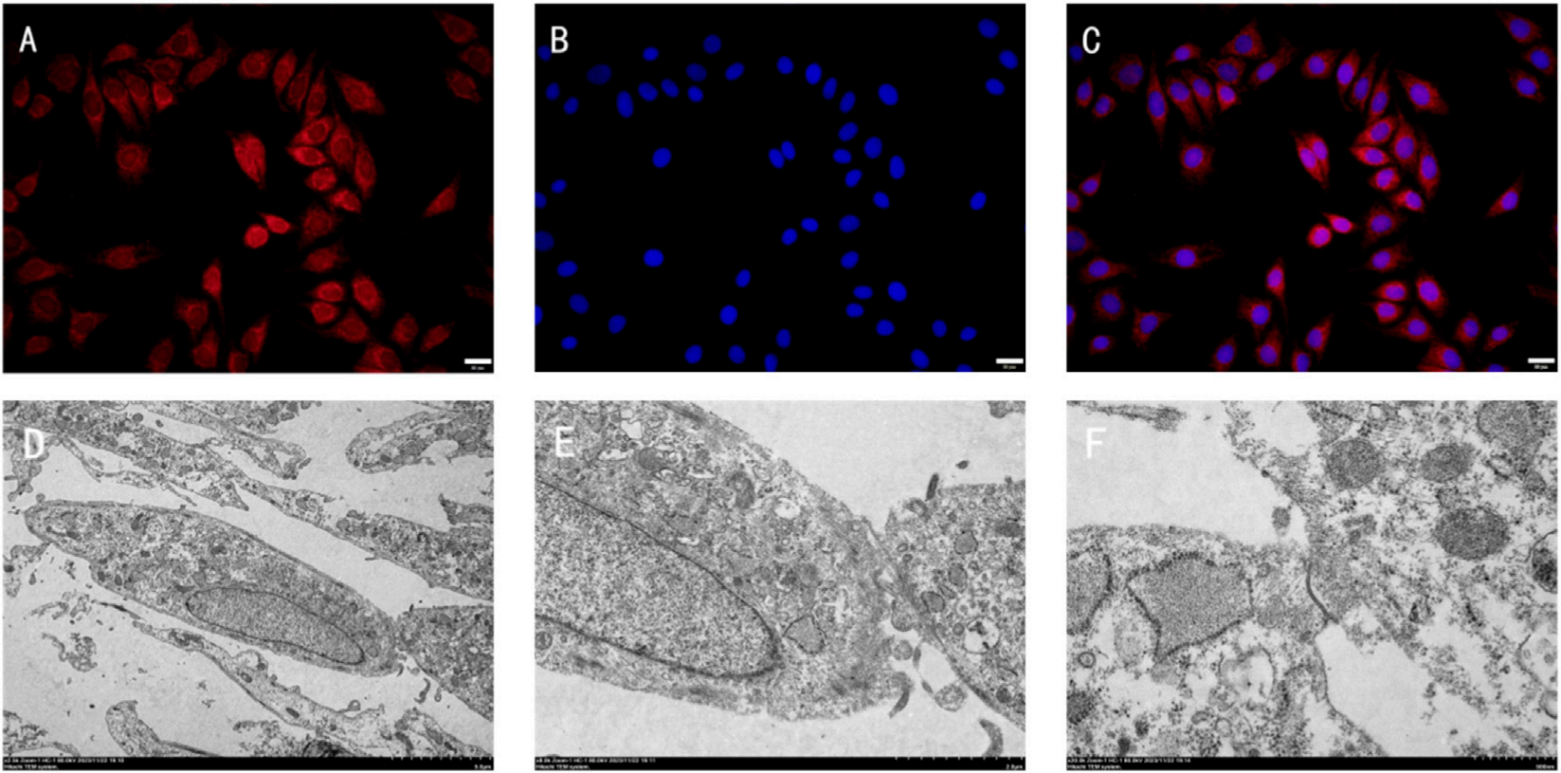
Immunofluorescence and TEM of urethral mucosal epithelial cells. **(A)** Cultured cells showing positive expression of pan-cytokeratin (PCK, red signal in the cytoplasm). **(B)** Nuclei counterstained with DAPI (blue). **(C)** Merged image of PCK and DAPI staining (×400). Scale bar = 20 μm. **(D)** TEM image (×2500). **(E)** TEM image (×8000). **(F)** TEM image (×20000) showing characteristic tonofilaments and desmosomal junctions (red arrows).

Cytokeratins are major components of the epithelial cytoskeleton and serve as specific markers for epithelial differentiation (Hickling et al., 2015). Immunofluorescence staining with an anti-PCK antibody confirmed the epithelial nature of the cultured cells, showing positive red cytoplasmic staining and DAPI-blue nuclei (Figures 3A–C). Transmission electron microscopy revealed distinct ultrastructural features, including abundant microvilli on the cell surface, normal cytoplasmic organelles, numerous tonofilaments, and large round nuclei with prominent nucleoli. Specialized intercellular junctions, such as tight junctions, desmosomes, adherens junctions, and gap junctions, were observed at cell membrane contact sites, indicating strong mechanical coupling. The cells exhibited robust proliferative capacity *in vitro* (Figures 3D–F).

### 3.2 *In Vitro* evaluation of cell–ADM constructs

**FIGURE 4:**
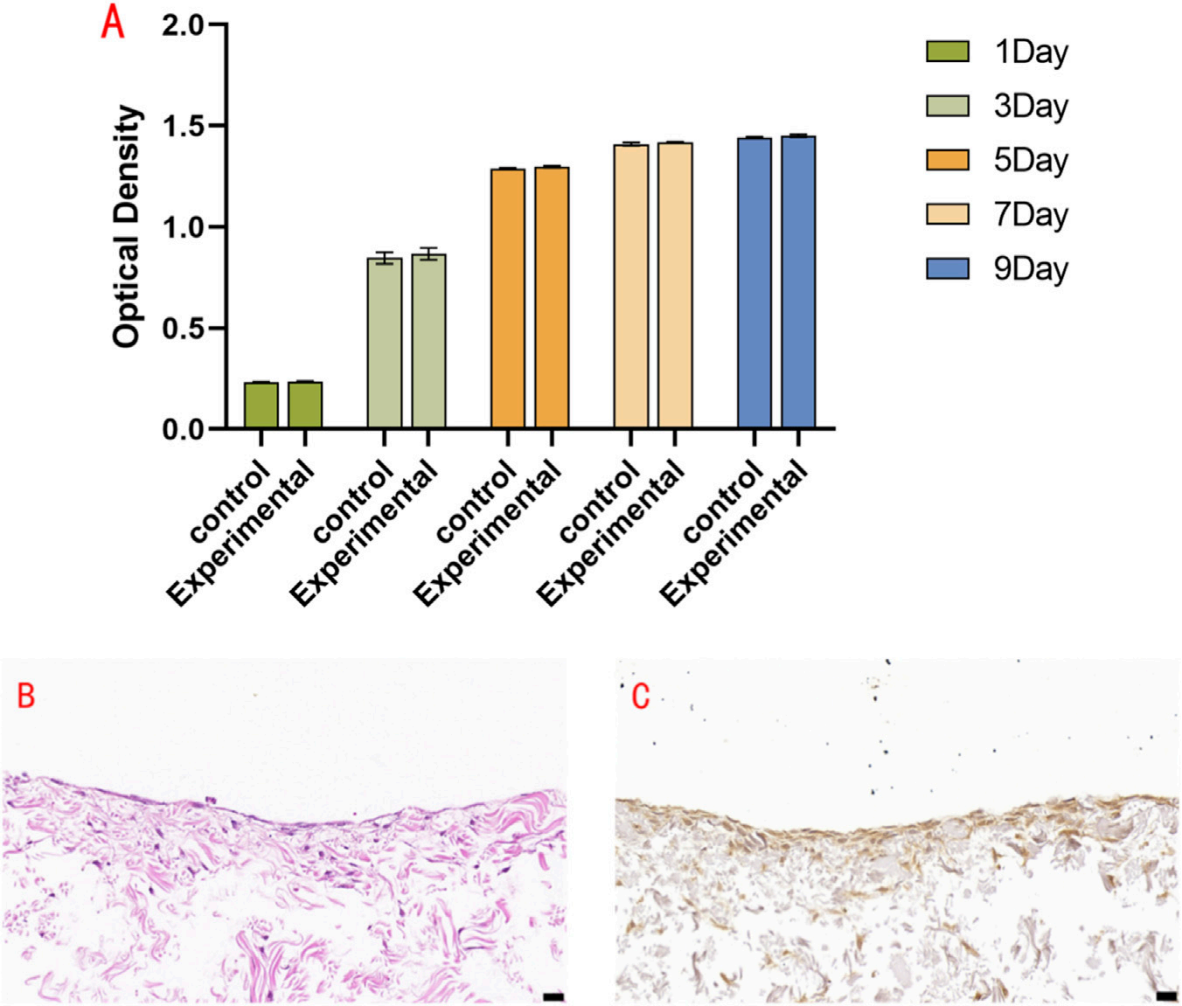
Cell–ADM biocompatibility and histological analysis. **(A)** CCK-8 assay showing no significant difference in cell proliferation between the control and experimental (cell-ADM) groups (P > 0.05), indicating favorable biocompatibility of the ADM scaffold. **(B)** H&E staining. **(C)** Immunohistochemistry for PCK (×400). Scale bar = 20 μm.

**FIGURE 5:**
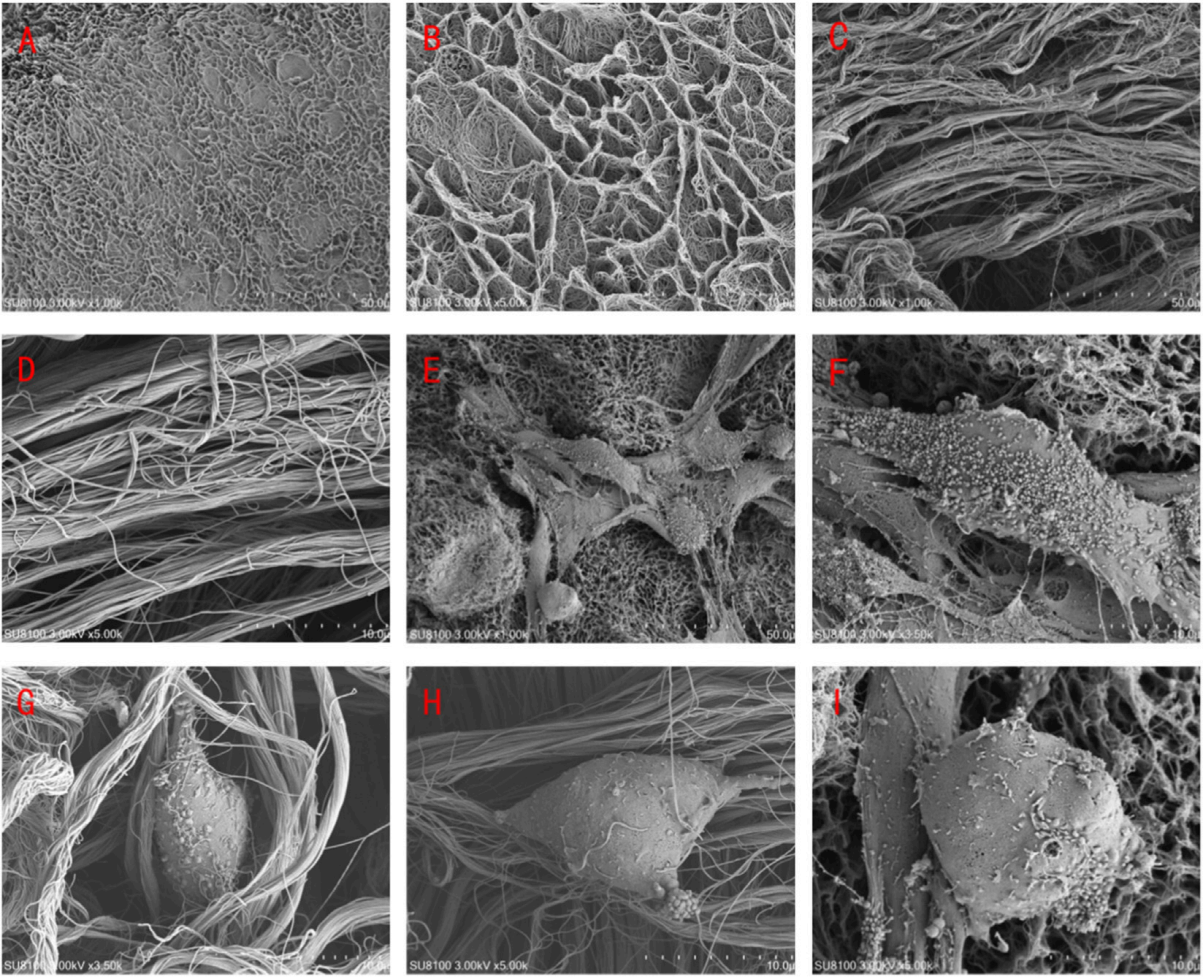
SEM analysis. **(A,B)** Surface view of the ADM scaffold at ×1000 and ×5000 magnification, respectively. **(C,D)** Cross-sectional view of the ADM scaffold at ×1000 and ×5000 magnification, respectively. **(E,F)** Surface view of the urethral mucosal epithelial cell–ADM co-culture at ×1000 and ×3500 magnification, respectively. **(G,H)** Cross-sectional view of the cell–ADM construct at ×3500 and ×5000 magnification, respectively. **(I)** High-magnification view (×5000) of urethral mucosal epithelial cells on the ADM scaffold.

The CCK-8 assay showed a time-dependent increase in cell viability in both the experimental (cell-ADM) and blank control groups, with no significant difference in proliferation kinetics (*P* > 0.05), indicating favorable ADM biocompatibility (Figure 4A). Histological examination showed that the urethral epithelial cells formed confluent sheets and developed a stratified structure of 2–3 layers on the ADM, which provides a functional barrier against urine penetration. Immunohistochemistry for PCK showed positive brownish-yellow cytoplasmic staining in the experimental group, with blue hematoxylin-counterstained nuclei, while the blank control was negative (Figures 4B,C). Scanning electron microscopy revealed a honeycomb-like structure on the ADM basement membrane surface. Urethral mucosal epithelial cells adhered to the ADM particles, exhibiting round or oval morphologies with microvilli and ridge-like cytoplasmic projections, and were surrounded by abundant extracellular matrix, indicating favorable adhesion, spreading, and growth (Figure 5).

### 3.3 *In Vivo* evaluation of tissue-engineered urethra

**FIGURE 6:**
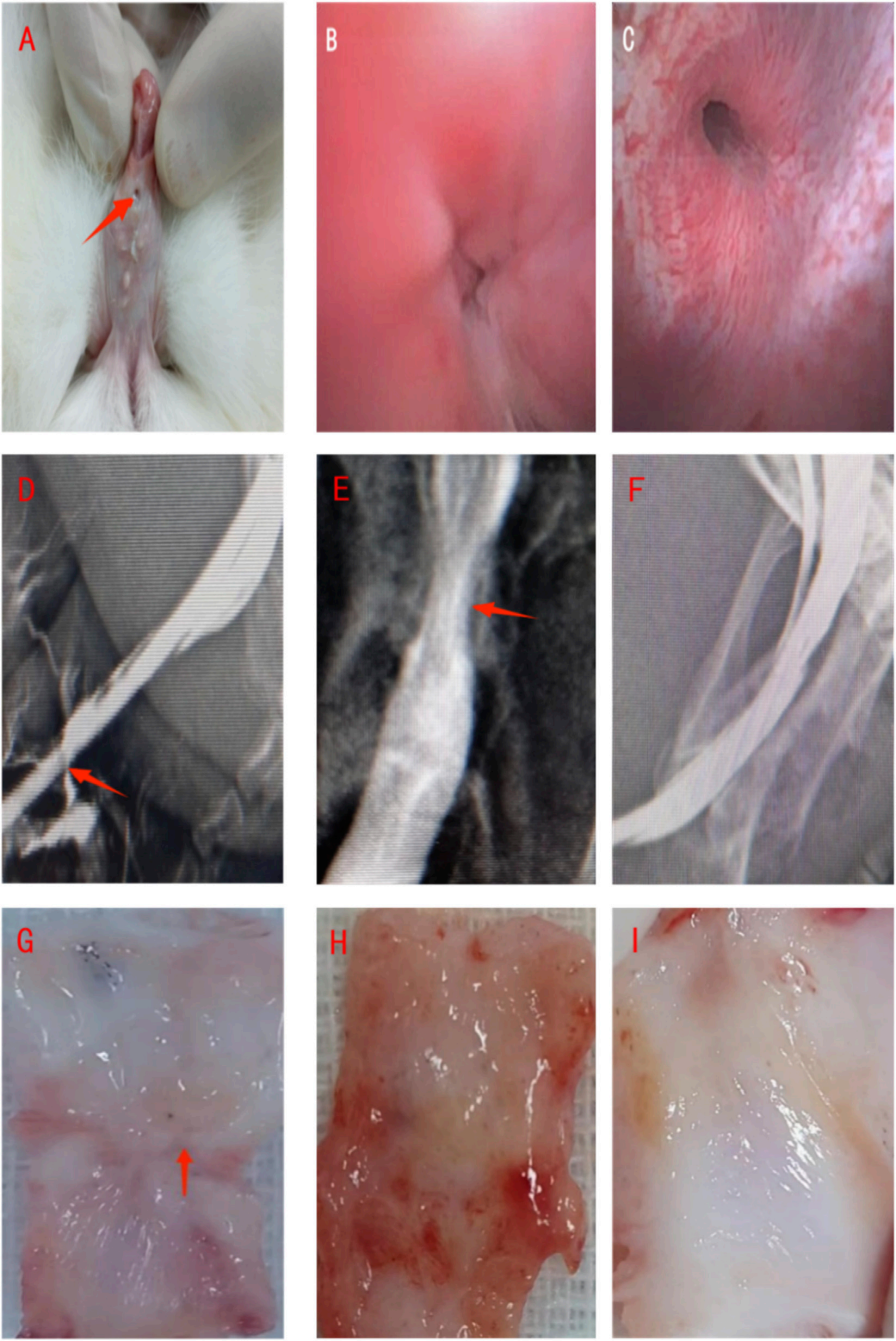
Urethroscopy, urethrography, and gross appearance of the tissue-engineered urethra at 12 weeks post-operation. **(A)** Gross view showing urethral fistula in Group A (red arrow). **(B,C)** Urethroscopic findings in Groups B and C, respectively. **(D–F)** Urethrography results: fistula formation in Group A (red arrow in D), urethral stricture in Group B (red arrow in E), and a patent urethral lumen in Group C **(F)**. **(G–I)** Gross morphological appearance of the urethra in Groups A, B, and C, respectively.

At 12 weeks post-operation, urethroscopy and urethrography were performed. In Group A, urinary fistula was observed (Figure 6A). In Group B, the scaffold was nearly completely absorbed, with a relatively smooth mucosal layer resembling native urethral tissue but with subtle textural differences; some cases exhibited fistula and stricture (Figure 6B). Group C showed near-complete scaffold absorption, a morphology indistinguishable from the native urethra, a smooth mucosal lining, and patent lumen (Figure 6C). Urethrography confirmed normal urethral contours in Group C without evident strictures. The complication rates were 100% in Group A (11 fistulas, 1 stricture), 66.67% in Group B (3 fistulas, 5 strictures), and 8.33% in Group C (1 fistula). Statistically significant differences were observed between Group A and Group B, as well as between Group A and Group C (*P* < 0.05). Furthermore, the difference between Group B and Group C was also statistically significant (*P* < 0.05) (Figures 6D–F; Table 2). Gross examination at 12 weeks revealed urethral scarring and contracture in Group B (Figure 6G, red arrow) and partial mucosal regeneration (Figure 6H). In contrast, Group C showed complete urethral mucosal regeneration, grossly indistinguishable from the native urethra (Figure 6I).

**TABLE 2:** Comparison of postoperative complications among groups A, B, and C.

	No	Yes	Incidence rate
Group A	0	12	100%#
Group B	4	8	66.67%#*
Group C	11	1	8.33%#*

#Group A showed significant differences compared to Groups B and C (*P* < 0.05); *Group B exhibited significant differences compared to Group C (*P* < 0.05).

**FIGURE 7:**
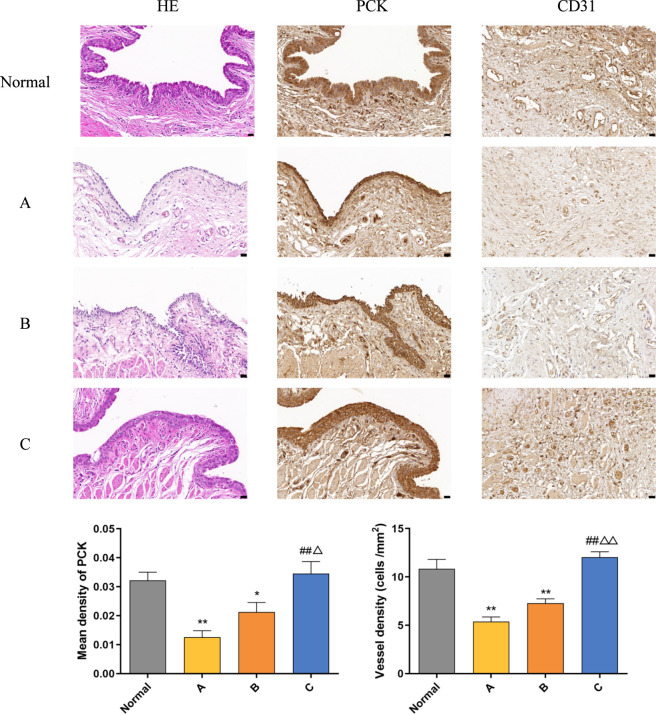
H&E and IHC (PCK and CD31) analysis of the normal group and post-operative groups **(A–C)** Scale bar = 20 μm. Data are expressed as mean ± SD (n = 3). *P < 0.05, **P < 0.01 vs. Normal group; #*P* < 0.05, ##*P* < 0.01 vs. Group A; △*P* < 0.05, △△*P* < 0.01 vs. Group B.

At 12 weeks post-operation, histological evaluation using H&E staining and IHC staining for PCK and CD31 expression revealed the following:Group A exhibited thin regenerated urethral mucosal epithelium with sparse capillary formation. Group B showed regenerated epithelial cells and capillaries, but with limited stratification.Group C demonstrated well-regenerated, multi-layered epithelium with clear stratification, a relatively dense and organized arrangement, and cuboidal cellular morphology resembling native urethral mucosa. Abundant smooth muscle bundles and a rich vascular network were observed. Quantitative analysis indicated that the expression levels of PCK (epithelial cells) and CD31 (vascular endothelial cells) in Groups A and B were significantly lower than those in the normal group (*P* < 0.05). In contrast, Group C exhibited expression levels of PCK and CD31 comparable to the normal group, with no statistically significant difference (*P* > 0.05). Furthermore, the expression in Group C was significantly higher than that in Groups A and B (*P* < 0.05) (Figure 7).

## 4 Discussion

Urethral stricture poses a significant clinical burden (Hampson et al., 2014; Wong et al., 2012). Although buccal mucosa graft urethroplasty remains the standard of care (Rashidbenam et al., 2019; Canning, 2005), it is associated with non-negligible donor site morbidity (Horiguchi, 2017; Chan et al., 2020). Tissue engineering has emerged as a promising strategy to overcome these limitations (Xue et al., 2016; Osman et al., 2015). While acellular scaffolds offer a straightforward approach (Palminteri et al., 2012; Versteegden et al., 2017), their success is highly dependent on a well-vascularized recipient bed and is often compromised by graft contraction and fibrosis in long-segment (>4 cm) defects (Casarin et al., 2022; De Filippo et al., 2002). In contrast, cell-seeded scaffolds demonstrate superior outcomes, with studies reporting a 5.7-fold higher long-term success rate compared to acellular grafts (Abbas et al., 2018), attributable to enhanced vascularization and the rapid establishment of a functional epithelial barrier (Abbas et al., 2019).

The selection of an appropriate cell source is paramount, guided primarily by the principle of “like-tissue regeneration” to construct a functional epithelial barrier (Xuan et al., 2022). Urothelial cells are inherently programmed to form a continuous, stratified squamous epithelium capable of withstanding the cytotoxic and inflammatory effects of urine. In comparison, cells from alternative sources, such as buccal mucosa, require adaptation to the urethral milieu. By employing homologous cells, we aim to achieve the most rapid and authentic restoration of this critical barrier, which is essential for preventing urine extravasation, subsequent inflammation, and fibrosis–key drivers of stricture recurrence. When seeded onto scaffolds and re-implanted, these cells are more likely to maintain their phenotype and function, secreting appropriate cytokines and extracellular matrix components to support the regeneration of underlying stromal tissue. Although obtaining these cells requires an invasive biopsy, presenting a clinical limitation, their biological advantages for urethral reconstruction are crucial and strongly justified our methodological choice in this proof-of-concept study. Emerging and increasingly validated techniques, such as harvesting urothelial cells via bladder washings or voided urine, offer a promising minimally invasive solution to this accessibility issue. These cells have demonstrated robust proliferative and differentiation potential *in vitro* (Fossum et al., 2003) and have been successfully utilized in clinical trials for urethral reconstruction (Fossum et al., 2007; de Kemp et al., 2015).

Tissue-engineered urethras constructed using seeded scaffold matrices can better mimic the native *in vivo* microenvironment, thereby enhancing the success rate of urethral defect repair and reducing complications (Jin et al., 2023; Jiang et al., 2024; Gundogdu et al., 2025). Our study further validates the feasibility of a tissue engineering approach utilizing autologous urothelial cells on an ADM scaffold. The substantial reduction in postoperative complications (8.33% in Group C versus 66.67% in the ADM-only group) underscores the pivotal role of seeded cells in promoting orderly tissue regeneration. This research demonstrates that the repair outcome of cell-ADM tubular constructs is significantly superior to ADM alone, with the fundamental innovation lying in the transition from “structural replacement” to “functional guidance.” Compared to the high failure rates reported in the literature for patch repair of long-segment strictures (Leng et al., 2025), the success of our tubular structure hinges not only on the provision of continuous physical support but, more critically, on the pre-seeded cells accelerating the formation of a continuous epithelial barrier and promoting intrinsic vascular network formation, which are key to achieving functional repair. Recent research in regenerative medicine similarly emphasizes that rapid epithelialization and effective angiogenesis are central to suppressing fibrosis, promoting normative tissue regeneration, and ensuring the long-term patency of tubular grafts (Yan et al., 2025).

Furthermore, this experiment involved the surgical creation of a 2.5 cm perineal tubular urethral defect in rabbits, repaired with the cell-seeded tissue-engineered ADM in a tubular configuration. Consequently, the surgical technique and operative environment directly influenced the experimental results. The corpus spongiosum is highly vascular. During surgery, exposing the tubular urethra requires dissection through the corpus spongiosum, and the complete resection of the tubular urethra inherently damages the spongiosal tissue, leading to significant hemorrhage. Therefore, a thorough understanding of rabbit urethral anatomy and proficient, standardized surgical technique are of paramount importance. Additionally, during the anastomosis of the cell-ADM tubular graft to the native urethral ends–a process where the scaffold and seeded cells transition from the culture medium into the host environment–the suturing process takes several minutes. It is imperative during this period to intermittently and gently irrigate the ADM with sterile culture medium using a syringe to prevent desiccation and ensure the viability of the seeded cells.

Our study, however, has several limitations. The use of a healthy rabbit model does not fully recapitulate the fibrotic and vasculodeficient environment characteristic of recurrent human urethral strictures. Regenerative capacity may be different under such pathological conditions. Moreover, the process of harvesting and expanding autologous urethral cells, while effective in this model, adds a layer of procedural complexity.

Future research efforts should focus on integrating truly non-invasive cell harvesting methods (e.g., via bladder washings) with advanced scaffold design. Exploring co-culture systems, combining epithelial cells with stem cells to further potentiate angiogenesis, represents a promising avenue for improving the outcomes of tubularized repairs in complex clinical scenarios.

## 5 Conclusion

For long-segment urethral defects, the tubularized cell-ADM composite emerges as a superior biological alternative. Our findings confirm that this construct not only supports the formation of anatomic and functional urethral tissue *in vivo* but also significantly outperforms its acellular counterpart, highlighting its translational potential as a viable strategy for complex urethral reconstruction.

## Data Availability

The original contributions presented in the study are included in the article/supplementary material, further inquiries can be directed to the corresponding author.
